# Association between gait and cognition in an elderly population based sample

**DOI:** 10.1016/j.gaitpost.2018.07.178

**Published:** 2018-09

**Authors:** Vyara Valkanova, Patrick Esser, Naiara Demnitz, Claire E. Sexton, Enikő Zsoldos, Abda Mahmood, Ludovica Griffanti, Mika Kivimäki, Archana Singh-Manoux, Helen Dawes, Klaus P. Ebmeier

**Affiliations:** a Department of Psychiatry, University of Oxford, Oxford, OX3 7JX, United Kingdom; b Movement Science Group, Oxford Brookes University, OX3 0BP, United Kingdom; c FMRIB Centre, Nuffield Department of Clinical Neurosciences, John Radcliffe Hospital, University of Oxford, OX3 9DU, United Kingdom; d Centre for Research in Epidemiology and Population Health, INSERM, U1018, Villejuif, France; e Department of Epidemiology and Public Health, University College London, United Kingdom

**Keywords:** Gait, Walking, Older adults, Healthy elderly, Cognition

## Abstract

**Background:**

Gait is thought to have a cognitive component, but the current evidence in healthy elderly is mixed. We studied the association between multiple gait and cognitive measures in a cohort of older people.

**Methods:**

One hundred and seventy-eight cognitively healthy participants from the Whitehall II Imaging Sub-study had a detailed clinical and neuropsychological assessment, as well as an MRI scan. Spatiotemporal and variability gait measures were derived from two 10 m walks at self-selected speed. We did a linear regression analysis, entering potential confounders with backwards elimination of variables with p ≥ 0.1. The remaining variables were then entered into a second regression before doing a stepwise analysis of cognitive measures, entering variables with p < 0.05 and removing those with p ≥ 0.1.

**Results:**

Amongst absolute gait measures, only greater stride length was associated with better performance on the Trail Making Test A (p = 0.023) and the Boston Naming Test (p = 0.042). The stride time variability was associated with performance on the Trail Making Test A (p = 0.031). Age was associated with poorer walking speed (p = 0.014) and stride time (p = 0.011), female sex with shorter stride time (p = 0.000) and shorter double stance (p = 0.005). Length of full-time education was associated with faster walking speed (p = 0.012) and shorter stride time (p = 0.045), and a history of muscular-skeletal disease with slower walking speed (p = 0.01) and shorter stride length (p = 0.015). Interestingly, volume of white matter hyperintensities (WMH) on FLAIR MRI images did not contribute independently to any of the gait measures (p > 0.05).

**Conclusions:**

No strong relationship between gait and non-motor cognition was observed in a cognitively healthy, high functioning sample of elderly. Nevertheless, we found some relationships with spatial, but not temporal gait which warrant further investigation. WMH made no independent contributionto gait.

## Highlights

• Gait is thought to have a cognitive component, but the evidence in elderly is mixed.
• We studied the association between multiple gait and cognitive measures.
• No strong relationship in a cognitively healthy, high functioning sample of elderly.
• Nevertheless we find some relationships with spatial, but not temporal gait.
• White matter hyperintensities made no independent contribution to gait measures.

## 1 Introduction

Gait disorders and cognitive impairment are common in people over the age of 65. However, there is considerable heterogeneity in cognitive ageing [1], and about 20% of even very old people walk well [2]. This implies that decline in cognition and mobility is not a necessary consequence of aging. Better understanding of gait and cognitive impairment is important in order to identify the drivers of successful ageing.

A recent review of epidemiological and neuropsychological evidence suggested that gait and cognition are closely related, but our knowledge of their interrelationship is limited [3]. Risk factors for decline in cognition and mobility may overlap, and possibly share pathological mechanisms including neurodegeneration, inflammation, and vascular damage [3]. There is evidence suggesting that gait measures are differentially related to distinct cognitive processes [[4], [5], [6]]. Abnormal gait has been linked to structural and functional brain changes, although the underlying neural networks are poorly understood, unlike the brain correlates of cognitive measures [6,7]. Studying the gait –cognition relationship can therefore provide further insights into shared cognitive and motor networks and identify targets for intervention [5,7,8]. Delaying cognitive and motor decline has huge public health implications as these two geriatric syndromes are associated with high healthcare costs and poor outcomes such as falls, disability and mortality [[9], [10], [11]].

A number of studies investigated the relationship between gait and cognition in healthy elderly, but their results are mixed [4]. The main focus has been on associations between executive function and gait, with limited evidence in other cognitive domains, such as memory, language, visuospatial ability or processing speed [4]. Although most population-based studies examined the associations between walking speed and cognitive function, relatively few focused on other gait variables [4,12]. Stride time variability, for example, may be a more sensitive indicator of early gait dysfunction and risk of falls than walking speed [13].

The purpose of this study was to investigate cognitive-motor interactions by examining the associations between a range of cognitive domains and a range of gait measures using quantitative gait analysis in a population-based sample of older people. Demnitz et al. also investigated the relationship between gait and cognition in this sample of older adults, but used as an outcome gait speed, measured with a stopwatch, from an earlier phase of the study (phase 9, 2007–2009), as well as balance and chair stands tests [14].

## 2 Methods

### 2.1 Participants

The Whitehall II study, an on-going, prospective cohort study, recruited 10 308 non-industrial civil servants across a range of employment grades in 1985–1988. They have been followed up at regular intervals [15]. Of the original 10 308 participants recruited in Phase 1, 6035 participated at the assessment in 2012–13. Eight hundred of these were randomly selected to take part in the Whitehall II Imaging Sub-study in 2012–2016. The detailed protocol of the study is described elsewhere [15]. This paper describes results from 241 participants, who also underwent gait assessment between November 2013 and January 2015. Participants were included, if they were willing and capable of giving informed consent, were able to complete cognitive testing and to walk unaided for 10 m. In addition to the general exclusion criteria in the Whitehall Imaging Sub-study such as contraindications to magnetic resonance imaging (MRI) scanning or being unable to travel to Oxford without assistance, participants were excluded from the current analyses if they had cognitive impairment (defined by MoCA score <26), a history of stroke or gross structural brain abnormality (e.g. a brain cyst or tumour) or missing cognitive and imaging data.

### 2.2 Procedure

The participants had a detailed clinical interview to confirm psychiatric and medical history, neuropsychological testing using validated instruments and an MRI scan [15]. The gait analysis was performed after their clinical and neuroimaging protocol. The Oxford Central University Research Ethics Committee approved the Whitehall II Imaging Sub-Study and informed written consent was given by all participants.

#### 2.2.1 Clinical assessment

General information such as age, sex, education, ethnicity, social class and full-scale IQ (FSIQ) was recorded for all participants, as well as information on alcohol intake, BMI, blood pressure, physical activity, depressive symptoms, medical history and current medications, as previously described [15]. Briefly, FSIQ was estimated from the Test of Premorbid Functioning (TOPF). Alcohol intake was recorded as average units of alcohol per week. BMI (kg/m2) was calculated, and participants were divided into three groups: normal weight (BMI 18.5–24.9), overweight (BMI 25–29.9) and obese (BMI > 30). Blood pressure was measured twice in a sitting position, after the cognitive protocol. Physical activity was measured using the Community Healthy Activities Model Program for Seniors (CHAMPS) questionnaire. Depressive symptoms were assessed using the Centre for Epidemiological Studies Depression Scale (CES-D) with a score of ≥16 representing clinically relevant depressive symptoms. History of neurological or musculoskeletal disease, according to ICD-10 Category VI or XIII was gathered by self-report. The number of prescribed drugs taken per day was recorded. MRI data were acquired at the Oxford Centre for Functional MRI of the Brain (FMRIB) using a 3-Tesla, Siemens Magnetom Verio scanner with 32-channel head coil. Details of acquisition sequences and pre-processing have been described previously [15]. White matter hyperintensities (WMH) were automatically segmented on FLAIR images with a newly developed tool, BIANCA (Brain Intensity Abnormality Classification Algorithm), a fully-automated, supervised method for WMH detection, based on the k-nearest neighbour algorithm [16]. Briefly, BIANCA classifies the image voxels based on their intensity and spatial features, where the intensity features were extracted from the images.

#### 2.2.2 Cognitive assessment

The following cognitive test battery was administered to all participants, as described previously [15]:

a) ***Montreal Cognitive Assessment*** (MoCA): visuospatial abilities, short-term memory, executive function, attention, working memory, language and orientation.
b) ***Trail Making Test version A and B*** both assess processing speed; version B adds a measure of cognitive flexibility, i.e. executive function.
c) ***Verbal fluency test: letter and category*** rely on set shifting and processing speed
d) ***Digit span***: ***forwards, backwards and sequence; the total score was included in the analysis –*** working memory
e) ***Digit Symbol substitution Test*** executive function and speed of processing
f) ***Rey-Osterrieth Complex Figure Test*** (RCF): immediate and delayed recall; visuo-spatial ability and visuospatial memory, but also attention, planning, and working memory
g) ***Hopkins Verbal Learning Test-Revised*** (HVLT-R): total recall; verbal learning and episodic memory
h) ***Boston Naming Test*** - confrontational word retrieval, language, visuo-perceptual skills
i) ***CANTAB reaction time***: simple and choice reaction time: processing speed and concentration

#### 2.2.3 Gait assessment

Participants performed two walks over a 10-m walkway free of obstacles at their self-selected walking pace. They started at a static position at the zero-point and came to a complete stop at the 10-m line. An inertial measurement unit (IMU) (MTX, Xsens, Netherlands), was placed over the projected centre of mass (CoM), located over the fourth lumbar vertebra [17], measuring at a sample frequency of 100 Hz.

We selected gait variables based on comparability with other population-based studies. The following absolute measures were used: walking speed (m/s, walking distance divided by the time taken to cover 10 m); stride length (m, distance between two successive placements of the same foot as derived via inverted pendulum methodology [18]; stride time (added left and right step time; step time was defined as the time interval between trough-to-trough CoM excursions during one gait cycle); and double stance (%, the percent of the total cycle during which the two feet are on the ground). We also measured three variability measures reported as coefficient of variation (CoV), namely CoV Walking speed, CoV Stride length and CoV Stride time.

### 2.3 Analysis

#### 2.3.1 Gait analysis

The IMU data were analysed using a custom program written in LabVIEW2015 (National Instruments, Ireland), to obtain vertical CoM excursion using IMU translatory acceleration in combination with previously published methods using quaternion rotation matrices and double integration [18]. Temporal parameters of gait, were derived from negative peak-to-peak CoM excursion and expressed in milliseconds [18]. Validated and well-established gait models were used to derive spatial gait parameters, reliant on leg length [19] and foot size [[20], [21], [22]] in relation to the vertical CoM excursion, resulting in derived stride length defined as the distance travelled from ipsilateral heelstrike-to-heelstrike. Relative variability of spatial parameters is defined as the coefficient of variation (CoV), defined as the ratio of the standard deviation to the average of stride time and length. Validation studies have been conducted on this approach in health [23,24] and clinical conditions [[25], [26], [27]] with and without gait abnormalities. An average of 13 steps, equalling 6 strides per 10 m-walk were recorded.

#### 2.3.2 Statistical analysis

Data were analysed using IBM SPSS version 24 for Windows. After inspection of data for normality, log-transform of the coefficient of variation measures (because of large positive skew), we did a linear regression analysis, entering potential confounders (age, sex, leg length, height, BMI, CES-D depression scores, FSIQ estimate from the Test of Premorbid Function (TOPF), a history of neurological or musculoskeletal disease, and volume of WMH as percentage of intracranial volume [28], eliminating variables successively with regression coefficients with p ≥ 0.1. The remaining variables were then entered into a second regression before doing a stepwise analysis of cognitive measures (MoCA, categorical fluency, letter fluency, Trail Making Test A and B, RCF immediate and delayed recall, HVLT immediate recall, Boston Naming Test, Digit recall sequence, and Digit symbol substitution test), entering variables successively with p < 0.05 and removing those with p ≥ 0.1. As these were exploratory analyses (n = 7; walking speed, stride length, stride time, double stance and log-transformed coefficients of variance of walking speed, stride length, and stride time as dependent variables), p-values < 0.05 were considered to indicate statistical significance.

## 3 Results

We examined the association between cognitive domains and gait parameters in a population-based sample of 241 older adults. After excluding participants with cognitive impairment (defined by MoCA score <26; n = 48), stroke (n = 2), gross structural brain abnormality on MRI scan (n = 7) and missing data (n = 6), the study sample consisted of 178 older people (mean age 69, SD 5.1). Their demographic and clinical characteristics are summarised in Table 1. The sample was predominantly male (75%), white (98%), middle class (76%), physically active and well educated with a median of 15 years of full-time education. A summary of the gait and cognitive measures is provided in Table 2.

**Table 1:** Demographic and clinical characteristics of the sample.

Variable	Summary
N	178
Age (y), mean (SD)	69 (5.1)
Male, n (%)	134 (75)
Ethnicity white, n (%)	175 (98)

Social class, n (%)	
Higher Middle Lower	29 (17) 130 (76) 12 (7)
Years of full-time education (y), median (IQR)	15 (5)
FSIQ (estimated from TOPF), mean (SD)	116.4 (6.9)
Height (m), mean (SD)	1.73 (0.1)
Weight (kg), mean (SD)	78.1 (13.8)
Leg length(cm), mean (SD)	95.1 (5.8)
BMI, median (IQR)	(4)

BMI, n (%)	
Normal (BMI 18.5-24.9) Overweight (BMI 25-29.9) Obese (BMI > 30)	84 (47) 67 (38) 27 (15)
Alcohol (units/week), median (IQR)	12 (14)
Systolic BP (mmHg), mean (SD)	140.5 (16.3)
Diastolic BP (mmHg), mean (SD)	75.4 (10.4)
CESD total score, median IQR	3 (5)
CESD cases cut off 15/16, n (%)	11 (5.9)
Number of current medication, median (IQR)	2 (3)
Weekly frequency of moderate exercise, median (IQR)	4 (7)
Weekly calorie expenditure in moderate exercise, median (IQR)	1262 (2094)
ICD-10 (VI) Nervous system disease, n (%)	17 (9.6)
ICD-10 (XIII) Musculoskeletal system and connective tissue disease, n (%)	66 (37)

**Table 2:** Summary of outcome measures.

Gait measure	
Walking speed (m/s), mean (SD)	1.5 (0.23)
Stride length (m), mean (SD)	1.56 (0.16)
Stride time (ms), mean (SD)	1080 (85)
Walking speed variability (m/s), mean (SD)	0.15 (0.16)
Stride length variability (cm), mean (SD)	0.10 (0.07)
Stride time variability (ms), mean (SD)	0.05 (0.05)
Double Stance (%),mean (SD)	27.6 (8.6)

Neuropsychological test	
MoCA total score, median (IQR)	28.5 (2)
Trail Making Test A (sec),^a^ mean (SD)	28.3 (8.4)
Trail Making Test B (sec),^a^ mean (SD)	63 (30)
Rey Complex Figure Immediate Recall, median (IQR)	18 (8)
Rey Complex Figure Delayed Recall, median (IQR)	17(8)
Category fluency, median (IQR)	23 (7)
Letter fluency, median (IQR)	16 (5)
Hopkins Verbal Learning Test total recall, median (IQR)	30 (5)
Boston Naming Test, median (IQR)	59 (2)
Digit span total, median (IQR)	31(8)
Digit Symbol Substitution Test, median (IQR)	64 (14)
CANTAB simple reaction time (ms),^a^ mean (SD)	303.2 (58)
CANTAB 5 choice reaction time (ms),^a^ mean (SD)	336.4 (48)

a Lower scores indicate better performance.

Effects of potential confounders and cognitive measures on gait measures (final models) are described in Table 3. The log-transformed coefficients of variation for walking speed and stride length were not associated with any of the confounders, nor any of the cognitive performance variables. Only the log-transformed coefficient of variation for stride time was associated with performance in the Trail Making Test A, in that greater variability of stride time was associated with longer time needed to complete the trails test. Amongst measures of gait, only stride length was associated with neuropsychology performance, in that greater stride length was associated with better performance in the Trail Making Test A and the Boston Naming Test. Of the confounders, age was associated with poorer walking speed and stride time, female sex with shorter stride time and shorter double stance. Length of full-time education was associated with faster walking speed and shorter stride time, and a history of muscular-skeletal disease with slower walking speed and shorter stride length. Interestingly, volume of WMH on FLAIR MRI images did not contribute independently to any of the gait measures.

**Table 3:** Effects of potential confounders and cognitive measures on gait measures (final models).

Model	Unstandardized Coefficients		Standardised Coefficients	t-value	p-value
	B	Std. Error	Beta		
Walking Speed					
(Constant)	0.923	0.361		2.560	0.011
Age	−0.008	0.003	−0.174	−2.494	0.014
Leg Length	0.010	0.003	0.247	3.552	0.000
Full-time Education	0.014	0.006	0.175	2.533	0.012
Musculoskeletal Disease	−0.061	0.026	−0.168	−2.398	0.01

Stride Length					
(Constant)	−0.151	0.293		−0.515	0.607
Sex	−0.039	0.025	−0.101	−1.566	0.119
Leg Length	0.014	0.002	0.516	8.009	0.000
Musculoskeletal Disease	−0.035	0.014	−0.144	−2.464	0.015
Trail Making Test A	−0.003	0.001	−0.135	−2.290	0.023
Boston Naming Test	0.009	0.004	0.126	2.053	0.042

Stride Time					
(Constant)	990.321	94.401		10.491	0.000
Age	3.060	1.191	0.185	2.569	0.011
Sex	−54.793	15.295	−0.264	−3.583	0.000
Full-time Education	−4.236	2.096	−0.147	−2.021	0.045
Musculoskeletal Disease	16.408	9.516	0.125	1.724	0.086

Duty Factor Double Stance					
(Constant)	70.653	20.367		3.469	0.001
Sex	−6.274	2.218	−0.297	−2.829	0.005
Height	−20.414	10.592	−0.202	−1.927	0.056

Natural Logarithm of Coefficient of Variance Stride Time					
(Constant)	−3.611	0.169		−21.353	0.000
Trail Making Test A	0.012	0.006	0.163	2.173	0.031

## 4 Discussion

### 4.1 Gait measures

Greater stride length was associated with faster processing speed and better performance on the Boston Naming Test. Few studies have investigated the association between processing speed and spatial gait parameters [8,29], but the results are inconsistent. They used different measures, limiting comparability. Interestingly, other studies also found associations between spatial parameters (e.g. *step length)* and cognitive measures such as executive function, rather than step time [8,29,30]. One hypothesis is that spatial measures relate to cortical areas, particularly the prefrontal cortex, while step time relies more on the brainstem and spinal cord [8]. With ageing the peripheral reflexes become less sensitive and there is less peripheral drive of gait with more reliance on central regulation [31]. Song and Geyer suggest feedback integration to be functionally more important than central pattern generation in human locomotion [32]. The reflexes drive the actions of calf muscles, which in turn drive gait and steps/strides. Thus, it is possible that early in ageing, the timing stays similar but spatial measures are affected.

We did not find any association between walking speed and cognitive measures. In contrast to our results, a previous larger study including this sample of older adults found that walking speed was associated with processing speed, category fluency and memory [14]. The discrepancy could be due to methodological differences: the two studies used different gait protocols, measured mobility at different time-points (i.e. years before the cognitive assessment vs. at the time of assessment) and had different exclusion criteria (e.g. we excluded participants with a MoCA<26). Importantly, due to smaller sample size, the current study may have lacked power to detect small differences.

Only three studies investigated the relationship between gait and language; they found association between walking speed and language, but did not specifically examine stride length [5,6,33]. The performance on the Boston Naming Test, is considerably affected by individual’s education, IQ and vocabulary [34,35]. This may have confounded the results, particularly considering that none of the other cognitive measures was associated with gait.

In summary, using absolute gait measures we see some relationships in the spatial, but not the temporal domain, and they may be the initial marker of change linked with cortical mechanisms.

### 4.2 Gait variability measures

Previous studies suggest that gait variability measures are foremost associated with executive function [6,8,[36], [37], [38]]. In this study, lower stride time variability was associated with processing speed (i.e. shorter time needed to complete the Trails A test), but against expectation there was no association between executive function and any gait variability measure.

### 4.3 General cognition

*None of the gait parameters* was significantly associated with general cognition (p > 0.05). In healthy older adults, a meta-analysis provided evidence of a small positive association between gait and global cognition (12 studies, d= 0.12, 95% CI = 0.09 to 0.15, *p* <.001) [12]. In this meta-analysis, speed of gait and MMSE were most commonly used. MMSE has lower sensitivity compared to MoCA [39], i.e. participants with wider range of cognitive abilities were included in the meta-analysis. In our study the cognitive scores showed very little variance (MoCA mean 28.3, SD 1.2), suggesting that a large sample would be required to detect differences and ceiling effects might have affected the results.

Overall, there is evidence to suggest that gait and cognition are related [3,4,12]. In a cognitively healthy and high-functioning sample of elderly, however, no strong relationship between gait and non-motor cognition was observed. As already mentioned, during ageing the peripheral sensory motor reflex loses sensitivity; this is associated with changes in connectivity resulting in enhanced brain activity during motor tasks [31,40]. When the coupling in the premotor-motor and peripheral sensory motor system is decreased, the prefrontal cortex compensates [40]. Michely et al. suggest that ageing results in U-shape change in premotor-motor connectivity, i.e. early in ageing connectivity increases, without additional requirement on the prefrontal structures; with advancing age premotor-motor connectivity starts to decline, increasing the demands on the prefrontal cortex. This model can explain the robust relationship found in studies with older participants [40].

It is also possible that he stronger relationship reported in studies with frailer participants was explained by the effect of common underlying brain pathology affecting cognitive and motor networks, rather than a direct link between gait and cognition. There are plausible pathophysiological mechanisms to explain this relationship, including neurodegeneration, inflammation and vascular damage; epidemiological, but also neuroimaging and intervention studies provide additional support [3]. Substantial methodological issues, however, limit the interpretation of the results. They are discussed below, highlighting the need for a robust methodological approach.

### 4.4 Methodological considerations and critical evaluation of research

The main strengths of the current study are that it was conducted in a large and well-characterised sample of cognitively healthy older adults. Also, participants underwent neuroimaging, a comprehensive neuropsychological testing and quantitative gait assessments providing temporal, spatial, as well as variability parameters. A sensitive test to screen for cognitive impairment was used, ensuring the detection of early cognitive changes. Analyses were controlled for multiple possible confounders, including not only socio-demographic variables, but also measures of physical, mental and brain health to exclude or control for potential pathological changes.

Limitations include the cross-sectional design, preventing us from making inferences about causality, lack of neurological examination and relying mainly on self-report to assess physical health. The current study only focused on a selected number of gait variables, while there are other aspects of movement not well characterised by our outcome measures. Also, our sample consisted of relatively younger, mostly male, high functioning, relatively active and well-educated elderly, limiting the generalizability of the findings.

### 4.5 Gait and health

It is well established that *gait is an indicator of health and general functioning* [41,42]. Gait disorders reflect age-associated diseases, rather than simply ageing [42]. They are multifactorial and to reliably study the relationship between gait and cognition a full physical examination, including neurological examination is essential. An assessment of disease severity and the detection of subclinical disease can provide important information as well.

Gait is a *multidimensional construct* and cannot be characterised by one parameter. Gait speed is a global marker of gait disturbance related to central, but also peripheral neuromuscular dysfunction and other gait measures, such as stride time variability are more specific correlates of cognitive measures [13,30]. On the other side it is important that the gait characteristics included in analysis are not redundant. Using factor analysis, a number of gait models have been proposed [[43], [44], [45]], but further research is required to confirm their validity. They differ in the number of suggested domains, e.g. three (pace, rhythm, variability) [44] versus five (adding asymmetry and postural control) [45]. More importantly, gait characteristics do not consistently load onto identical domains; for example, stride time variability can represent the variability [44] or pace [45] domain.

Gait has also been measured using different protocols with limiting comparability. In this study, a self-selected speed and simple gait were used. Walking at a faster speed or in more challenging environment imposes higher demands on postural and cognitive control. Previous studies have found a differential effect of fast versus self-selected walking speed on cognition [46,47]. A PET study demonstrated an association between increased complexity of gait such as obstacle avoidance and increased cortical activity [48].

### 4.6 Cognition and health

Studies classified as investigating gait and cognition in healthy elderly, *included participants with cognitive impairment*. The MMSE was the most common screening tool (cut off <24) [12] and only participants with dementia were excluded. In some studies, an MMSE cut off of 18 was used [46], participants who scored 0/1 on the MMSE delayed recall were included [49], 27% of the sample had impaired MMSE score (mean, SD 20.97 ± 2.97) (50) or general cognition was not assessed at all [38].

MMSE has poor sensitivity to detect mild cognitive impairment (MCI), due to a lack of complexity, dependence on demographic factors and inadequate assessment of executive and visuospatial function [39]. Ninety-eight percent of people diagnosed with MCI scored ≥24 on MMSE [39], and executive dysfunction was found to be common amongst people with a normal MMSE [51]. In contrast, MOCA (cut off of <26) has 90% sensitivity for detecting early cognitive changes [52]. To our knowledge, only four studies have used the MoCA, and they included participants with MoCA <26 [14,[53], [54], [55]]. Considering that gait is impaired in MCI compared to normal ageing [44], including participants with cognitive impairment is likely to affect the results significantly.

Similar to gait, cognition is a *multifaceted construct*. Neuropsychological tests overlap in the cognitive domains they measure. We did not group the tests, as the same tests were classified as measuring different domains, creating ambiguity. For example, category fluency is defined as representing executive function [8,12] or language [41] and digit span forward as reflecting attention/processing speed [56] or executive function [8,12]. Further, as cognitive domains are interdependent, it is difficult to assess their individual integrity without measuring performance in each domain. For instance, impaired attention will affect performance not only on executive function/attention measures, but also memory. To understand better the independent contribution of cognitive domains to gait, a comprehensive battery of neuropsychological tests assessing all domains should be used.

### 4.7 Clinical implications

Although there are associations between gait and cognition it is necessary to address a number of questions before quantitative gait assessment can become clinically useful. First, we need to specify which covariates and confounders are relevant, as they need to be matched between controls and patients. Second, we need to establish not only a difference in mean measures, but also compare different cut-offs for gait performance using receiver-operating characteristic (ROC) curves to optimise sensitivity and specificity. Finally, we hope future research can associate particular aspects of gait impairment with specific causes of brain diseases or relevant mechanisms. If these conditions are fulfilled, quantitative gait analysis as described could become a useful tool to complement clinical assessment.

## 5 Conclusions

No strong relationship between gait and non-motor cognition was observed in a cognitively healthy, high functioning population-based sample of elderly. Interestingly, we found some relationships in the spatial, but not the temporal domain, and they may be the initial marker of change linked with cortical mechanisms. WMH made no independent contribution to gait measures in this healthy older sample.

Understanding the relationship between spatial gait and cognition better, as well as the underlying pathophysiological mechanisms, has the potential to aid the prevention and treatment of cognitive and gait disorders [3]. Considering the significant public health implications of delaying cognitive and motor decline, further research is warranted. Substantial methodological issues hinder the advance in this area of research, highlighting the need for a robust methodological approach. It appears that in order to confirm such weak associations in healthy ageing volunteers, larger samples are required.
